# Targeting autophagy as a therapeutic strategy against pancreatic cancer

**DOI:** 10.1007/s00535-022-01889-1

**Published:** 2022-06-21

**Authors:** Keisuke Yamamoto, Dosuke Iwadate, Hiroyuki Kato, Yousuke Nakai, Keisuke Tateishi, Mitsuhiro Fujishiro

**Affiliations:** Department of Gastroenterology, Graduate School of Medicine, The University of Tokyo, 7-3-1 Hongo, Bunkyo-ku, Tokyo 113-8655 Japan

**Keywords:** PDAC, Autophagy, Lysosome, Host autophagy, Anti-tumor immunity

## Abstract

Macroautophagy (hereafter autophagy) is a catabolic process through which cytosolic components are captured in the autophagosome and degraded in the lysosome. Autophagy plays two major roles: nutrient recycling under starvation or stress conditions and maintenance of cellular homeostasis by removing the damaged organelles or protein aggregates. In established cancer cells, autophagy-mediated nutrient recycling promotes tumor progression, whereas in normal/premalignant cells, autophagy suppresses tumor initiation by eliminating the oncogenic/harmful molecules. Pancreatic ductal adenocarcinoma (PDAC) is a deadly disease that is refractory to most currently available treatment modalities, including immune checkpoint blockade and molecular-targeted therapy. One prominent feature of PDAC is its constitutively active and elevated autophagy-lysosome function, which enables PDAC to thrive in its nutrient-scarce tumor microenvironment. In addition to metabolic support, autophagy promotes PDAC progression in a metabolism-independent manner by conferring resistance to therapeutic treatment or facilitating immune evasion. Besides to cell-autonomous autophagy in cancer cells, host autophagy (autophagy in non-cancer cells) supports PDAC progression, further highlighting autophagy as a promising therapeutic target in PDAC. Based on a growing list of compelling preclinical evidence, there are numerous ongoing clinical trials targeting the autophagy-lysosome pathway in PDAC. Given the multifaceted and context-dependent roles of autophagy in both cancer cells and normal host cells, a deeper understanding of the mechanisms underlying the tumor-promoting roles of autophagy as well as of the consequences of autophagy inhibition is necessary for the development of autophagy inhibition-based therapies against PDAC.

## Background

Pancreatic ductal adenocarcinoma (PDAC) is one of the most lethal malignancies, with a 5-year survival rate of just above 10% [1] due to the lack of early detection methods and effective treatment regimens. Despite the unprecedented success of immune checkpoint blockade (ICB) and molecular-targeted therapy in other types of cancer, the vast majority of patients with PDAC have failed to benefit from these treatment methods, apart from a minority of patients with microsatellite instability or *KRAS-G12C* mutation, features associated with a better response to ICB [2–4] or KRAS-G12C-specific inhibitors [5]. Major genetic alterations in PDAC include activating mutations in the *KRAS* oncogene, which occur early during carcinogenesis in more than 90% of patients with PDAC, and inactivating mutations in tumor suppressor genes, such as *TP53*, *SMAD4*, and *CDKN2A*, which occur at later stages of disease progression in 60–70%, > 50%, and > 50% patients, respectively [6]. Unfortunately, these major mutations in PDAC remain undruggable. Therefore, it is imperative to identify novel therapeutic targets for this deadly disease based on its biological features.

**Fig. 1:**
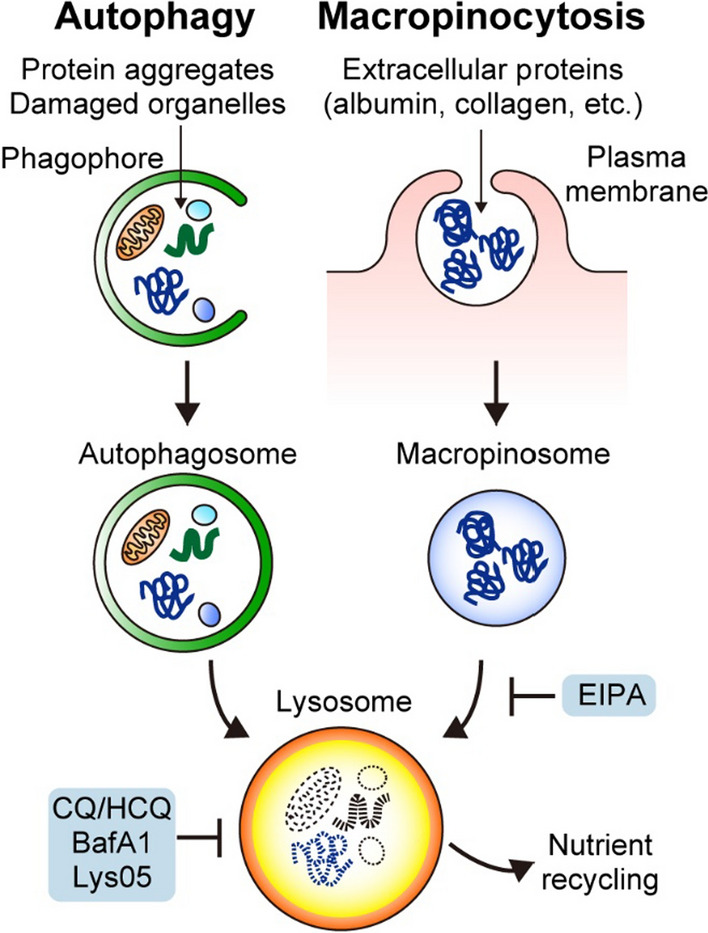
Pancreatic ductal adenocarcinoma (PDAC) relies on autophagy and macropinocytosis for nutrient scavenging. PDAC cells show elevated autophagy and macropinocytosis. Autophagy targets intracellular constituents, such as protein aggregates, damaged organelles, and lipids, whereas macropinocytosis enables bulk uptake of extracellular proteins, such as serum albumin or collagen, in the tumor microenvironment. Autophagy captures its cargo with double-membrane vesicles, termed autophagosomes, while macropinocytosis engulfs a portion of extracellular fluids and materials via invagination of the plasma membrane and formation of single-membrane vesicles, termed macropinosomes. Both autophagosomes and macropinosomes are fused with lysosomes for the degradation of cargo and recycling of nutrients. Inhibitors of these pathways are shown. *EIPA* 5'-(N-ethyl-N-isopropyl)amiloride, *CQ* chloroquine, *HCQ* hydroxychloroquine, *BafA1* bafilomycin A1

One characteristic feature of PDAC tumor is its dense fibrotic stromal component, termed desmoplasia, which is composed of extracellular matrix and various stromal cells, including fibroblasts and immune cells [7]. This dense stroma hampers the efficient delivery and diffusion of oxygen and nutrients within the tumor. In addition, nutrient competition among different cell types within the tumor microenvironment (TME) makes it even harder for PDAC cells to obtain nutrients and oxygen [8]. Indeed, PDAC is one of the most hypoxic tumors [9, 10], and nutrients are scarce in surgically resected human and murine PDAC tumors as compared with normal pancreas [11, 12]. To sustain and maximize proliferation in this harsh TME, PDAC relies on enhanced intracellular scavenging via autophagy [13], extracellular scavenging via macropinocytosis [14] (Fig. 1), and nutrient acquisition from normal cells in the TME [8], all of which represent potential therapeutic targets.

**Fig. 2:**
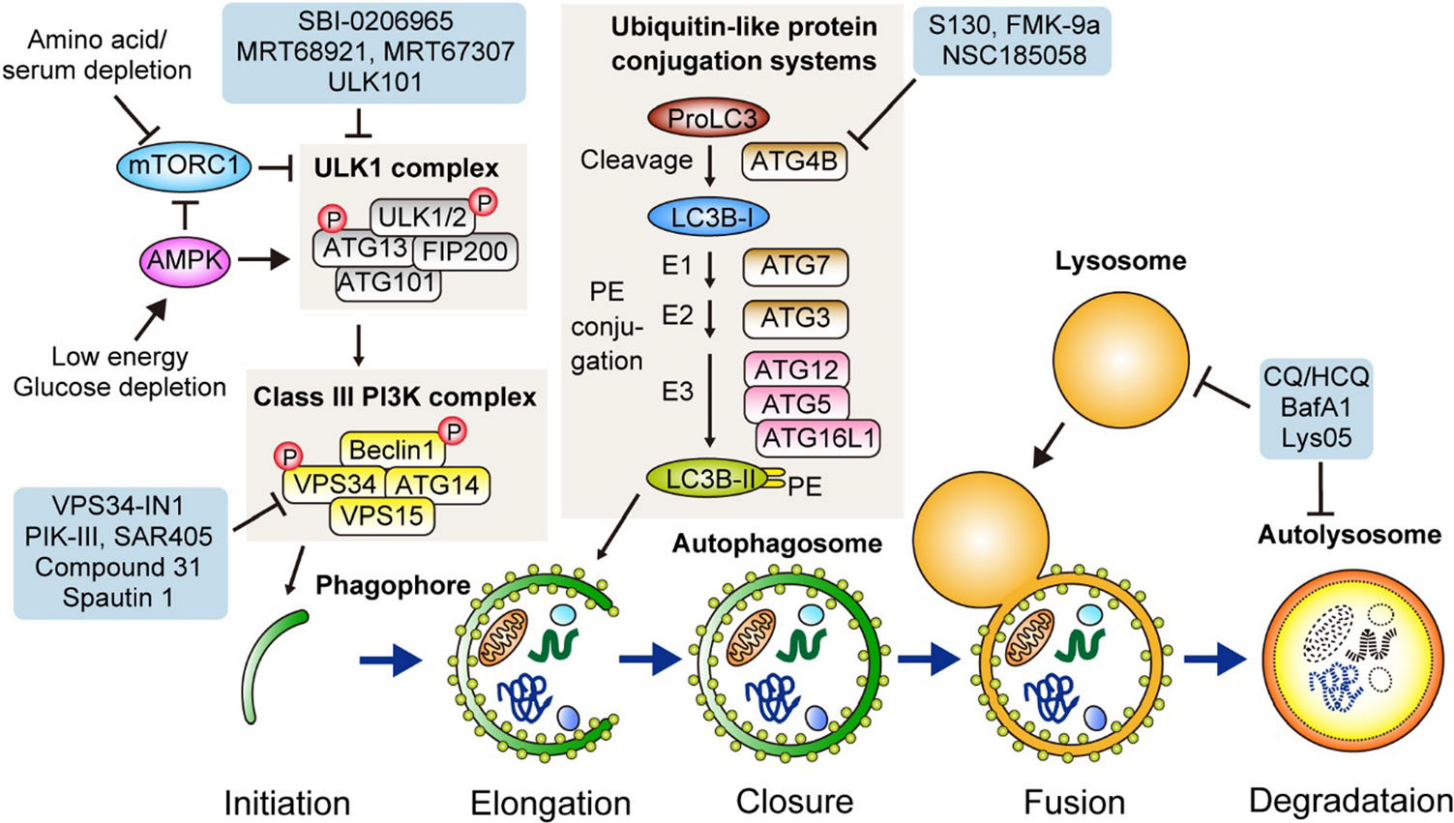
Overview of the general autophagy pathway in mammalian cells. (Bottom) Autophagy can be divided into five major steps: (1) Initiation and nucleation of the double-membrane phagophore, (2) elongation and (3) closure of the phagophore to form the autophagosome, (4) autophagosome-lysosome fusion, and (5) lysosomal degradation and nutrient recycling. (Top left) Autophagy induction is primarily mediated by the ULK1 complex, which is regulated by AMPK and mTORC1. Upon activation, the ULK1 complex activates the class III phosphatidylinositol 3-kinase (PI3K) complex through phosphorylation of beclin 1 (BECN1) and VPS34. The Class III PI3K complex generates PI3P at the site of nucleation of phagophore from ER. (Top middle) ProLC3B is converted to LC3B-I via the cleavage by ATG4B. LC3B-I is conjugated with PE through ubiquitin-like conjugation systems that include ATG7 (E1 ligase), ATG3 (E2 ligase), and ATG12, ATG5, and ATG16L (the E3 ligase complex). The resulting PE-conjugated LC3, which is called LC3B-II (shown as small green circles), is inserted on the phagophore membranes, where it facilitates phagophore elongation and closure. (Pale blue frames) Inhibitors of ULK1/2 (SBI-0206965 [139], MRT68921, MRT 67,307 [140], ULK101 [141]), VPS34 (VPS34-In1 [142], PIK-III [143], SAR405 [144], Compound 31 [145], Spautin1 [146]), ATG4B (S130 [147], FMK-9a [148], NSC185058 [149]), and the lysosome [CQ/HCQ, BafA1, Lys05 [150]]) are shown. *ULK* Unc-51-like kinase, *PI3K* phosphatidylinositol 3-kinase, *PI3P* phosphatidylinositol 3-phosphate, *ER* endoplasmic reticulum, *WIPI* WD-repeat protein interacting with phosphoinositide, *mTORC1* mammalian target of rapamycin complex 1, *AMPK* 5' AMP-activated protein kinase, *PE* phosphatidylethanolamine, *p62/SQSTM1* sequestosome 1, *NBR1* neighbor of BRCA1

Macroautophagy (hereafter autophagy) is a multistep catabolic/self-degrading process in which intracellular components are captured by the autophagosomes and degraded in the lysosomes (Fig. 2). The breakthrough discovery of autophagy-related genes by Dr. Ohsumi, who received the Nobel Prize in Medicine in 2016, has accelerated our understanding of the molecular mechanisms and roles of autophagy in both physiological and pathological contexts. Autophagy plays two key roles: (1) nutrient recycling and (2) maintenance of cellular homeostasis [15–18]. The nutrient-recycling role of autophagy is mediated mainly via bulk/non-selective autophagy, in which autophagy substrates are randomly captured and degraded. In contrast, the homeostatic role is mediated mainly via selective autophagy, in which specific targets, such as misfolded proteins, protein aggregates, or damaged organelles, are selectively removed with the help of autophagy cargo receptor proteins that recognize and bind to their cargo to facilitate trafficking to autophagosomes [15, 17, 19]. Thus, dysfunction of selective autophagy leads to the accumulation of undesired cargo, ultimately leading to diseases, such as neurological degenerative disorders and cancer [17, 18]. Generally, bulk autophagy is induced under stress conditions, such as nutrient starvation and hypoxia, while selective autophagy is mediated by basal autophagy, which is constitutively active at the basal level regardless of nutrient status.

The role of autophagy in cancer is complicated and highly context-dependent; however, in general, the nutrient-recycling activity of autophagy promotes tumor growth by fueling tumor metabolism, whereas the homeostatic role, mainly mediated by selective autophagy, prevents tumor initiation by removing oncoproteins or potentially carcinogenic cellular constituents in normal or precancerous cells [17]. As nutrient acquisition is almost always a limiting factor for cancer-cell proliferation, autophagy-mediated nutrient recycling plays a significant role in supporting tumor growth [20, 21]. Indeed, autophagy inhibition in established tumors suppresses tumor growth in various types of cancer, especially those driven by oncogenic mutations in *RAS* or *RAF*, such as lung cancer, pancreatic cancer, and melanoma, where autophagy is constitutively activated at high levels [13, 22–27].

PDAC is a cancer with constitutively active autophagy and increased lysosomal activity/function, facilitating its sustained growth in nutrient-scarce microenvironments. Besides this metabolic support via nutrient recycling, autophagy also promotes the progression of PDAC and other cancers via non-metabolic functions, such as treatment resistance or immune evasion. More recently, in addition to autophagy in cancer cells, host autophagy (autophagy in normal host cells) has been shown to play an important role in promoting tumor progression. In this review, we have highlighted the current knowledge on the roles and mechanisms of autophagy in cancer biology, with a special focus on PDAC.

### Autophagy suppresses the formation of precancerous lesions, but promotes their progression to cancer

Loss of essential autophagy genes in specific tissues causes various degenerative and inflammatory diseases in mouse models, reflecting the homeostatic role of autophagy in normal tissues [17]. Similarly, autophagy deficiency is implicated in tumorigenesis in several organs, including the liver, lung, and pancreas [23, 28–32]. The link between autophagy and carcinogenesis was first described in mouse models, where heterozygous knockout of the autophagy-related gene, beclin 1 (*Becn1*), increased the incidence of liver and lung tumors [33, 34], which is in line with frequent allelic loss of *BECN1* in human breast, ovary, and prostate cancers [35, 36]. However, follow-up studies suggested that *BECN1* could be a passenger gene of the neighboring tumor suppressor gene, *BRCA1* [37], raising concerns about the roles of *BECN1* and autophagy as tumor suppressors.

In 2011, two groups independently showed that mosaic deletion of autophagy-related (*Atg*)-*5* in the whole body or liver-specific deletion of *Atg7* leads to spontaneous formation of adenoma, a benign tumor, in the mouse liver [28, 29], confirming the role of autophagy as a suppressor of tumorigenesis. Similarly, *Atg5* ablation in KRAS-driven mouse lung cancer models accelerated benign tumor formation, but restricted its progression to adenocarcinoma [23, 30]. Likewise, studies using genetically engineered mouse models (GEMMs) of PDAC (Pdx-Cre; LSL-Kras^G12D/+^;Trp53^+/+^ or Trp53^flox/+^) have shown that in the presence of oncogenic Kras, autophagy deficiency accelerates the formation of premalignant pancreatic intraepithelial lesions (PanINs), but blocks the progression of these benign lesions to a malignant state (PDAC) [31, 32, 38]. In the absence of oncogenic Kras, autophagy deletion alone does not lead to PanIN formation [32, 38, 39]. Notably, in a similar autochthonous KRAS-driven pancreatic cancer model with homozygous p53 deletion (Pdx1-Cre; LSL-Kras^G12D/+^;Trp53^flox/flox^), *Atg5* or *Atg7* knockout accelerated tumor growth [31]. These paradoxical results may be explained by the non-physiological situation in the latter model where both copies of Trp53 were simultaneously lost during embryogenesis and the pancreas developed without ever expressing p53, which may have had an immense impact on normal organogenesis and tumorigenesis [40], while in the heterozygous p53 knockout model, Trp53 was lost in a step-wise manner during tumorigenesis, which more faithfully recapitulates human tumors [32]. Together, these results suggest that autophagy plays a dual role in carcinogenesis, blocking the initiation of benign premalignant lesions and promoting their progression to malignant cancer.

One possible mechanism for enhanced carcinogenesis following autophagy ablation is the pathogenic accumulation of autophagy cargo receptor proteins, which are normally degraded via the autophagy-lysosome system, together with their cargo. As most autophagy cargo receptor proteins have multiple functions and act as signaling molecules [41], their accumulation in autophagy-deficient cells can promote tumor formation and progression via multiple mechanisms, including aberrant signal activation [42, 43]. For example, *Atg5* or *Atg7* deletion in mouse liver accelerates adenoma formation [28, 29]. Mechanistically, accumulation of sequestosome 1 (SQSTM1/p62) in autophagy-deficient hepatocytes leads to the activation of the nuclear factor-erythroid 2-related factor 2 (NRF2) signaling, a central pathway regulating the redox and stress response genes [44], by derepressing NRF2 from Keap1-mediated regulation, thereby promoting adenoma development [29, 45–47]. Importantly, p62 is a major component of Mallory-Denk bodies [48], which are frequently observed in chronic liver diseases and hepatocellular carcinoma [29, 47], further supporting the role of p62 accumulation in liver tumorigenesis.

In addition to bulk autophagy, selective autophagy also plays an important role in both physiological and pathological conditions, although the role of selective autophagy in PDAC development remains less studied than that of bulk autophagy. Mitophagy is a form of selective autophagy in which damaged mitochondria are removed either via a ubiquitin-dependent pathway, such as the PINK1/Parkin pathway, or a ubiquitin-independent pathway that is mediated by the mitophagy receptors, BNIP3, BNIP3L/NIX, FUNDC1, and Bcl2L13 [49]. Interestingly, oncogenic *Kras* upregulates Nix-mediated mitophagy and removes functional mitochondria, thereby limiting glucose flux to the mitochondria and enhancing the redox capacity to promote PanIN progression to PDAC. *Nix* deletion increases mitochondrial mass and reactive oxygen species (ROS) production in PanIN cells, blocking PanIN progression to PDAC [50]. In contrast, PINK1/Parkin-mediated mitophagy has been reported to inhibit pancreatic tumorigenesis by regulating the local immune response [51]. Given that both NIX and PINK1/Parkin have mitophagy-independent roles and functions, further studies are required to elucidate the exact role of mitophagy in PDAC carcinogenesis.

### PDAC relies on constitutively active autophagy for sustained tumor growth

As mentioned previously, autophagy inhibition in established tumors suppresses tumor growth in multiple types of cancer, including PDAC, lung cancer, prostate cancer, and melanoma [13, 22–26, 52]. In 2011, human PDAC tumors and cell lines were shown to exhibit elevated levels of basal autophagy, and pharmacological or genetic inhibition of autophagy slowed the tumor growth via impaired mitochondrial metabolism and ROS accumulation [13]. This was further confirmed in a GEMM of PDAC that is driven by *KRAS* mutation and p53 loss of heterozygosity (Pdx1-Cre + ; LSL-Kras^G12D/+^; Trp53^flox/+^), where biallelic deletion of *Atg5* decreased PDAC formation and prolonged the survival of mice [32]. Atg5-null tumors exhibit impaired proliferation, increased DNA damage, and apoptosis [32]. Importantly, pharmacological inhibition of autophagy by the lysosome inhibitor, hydroxychloroquine (HCQ), exerts tumor-suppressive effects on murine PDAC cell lines and a panel of human patient-derived xenografts, regardless of their Trp53 status [32]. Notably, a more recent study using PDAC GEMM (KRAS mutation and wild-type Trp53) (Ptf1a-Cre + ; LSL-Kras^G12D/+^) showed that monoallelic loss of *Atg5* unexpectedly increased metastasis and shortened the survival of mice [53], although biallelic loss of Atg5 reduced PDAC formation, consistent with previous reports [31, 32]. However, it is unclear whether this enhanced aggressiveness of PDAC with monoallelic loss of Atg5 is a consequence of autophagy deficiency or autophagy-independent functions of Atg5, given that most autophagy-related proteins play multiple roles that are independent of autophagy [54].

In addition to these conditional knockout models, several new models have been developed to better understand the roles of autophagy at different stages (such as normal/premalignant lesions or full-blown tumors) and in different cell types (such as malignant or normal host cells) [26, 38, 55, 56] and to model autophagy inhibitor treatment strategies in patients. Yang et al. developed Kras-driven PDAC GEMMs, in which autophagy can be inhibited by doxycycline-inducible expression of Atg4B^C74A^, a dominant negative form of the Atg4B mutant that potently inhibits autophagy [38, 57]. Once PDAC tumors were formed, autophagy was inhibited by doxycycline treatment. Upon doxycycline treatment, PDAC tumors carrying homozygous Atg4B^C74A^ alleles showed rapid tumor regression, confirming the essential role of autophagy in established PDAC tumors. Karsli-Uzunbas et al. developed a murine non-small lung cancer model driven by Kras^G12D^ and Trp53 deletion, where Atg7 can be acutely deleted in the whole body in a tamoxifen-inducible manner (Ubc-CreERT2^/+^; Atg7^flox/flox^) [26]. When Atg7 was deleted after the formation of lung tumors, the tumors showed drastic regression [26]. Importantly, both models allow the inhibition of autophagy separately in cancer cells, normal host cells, or both, which revealed that blocking autophagy in normal host cells alone has a negative impact on tumor progression, shedding light on the role of host autophagy, which will be discussed later.

### Mechanism of enhanced autophagy-lysosome function in PDAC

Autophagy is induced under various stresses, such as starvation, hypoxia, organelle damage, DNA damage, endoplasmic reticulum (ER) stress, and pathogen infection [17]. Autophagy induction in response to nutritional changes is primarily mediated by the unc-51 like autophagy activating kinase 1 (ULK1) complex, which is positively and negatively regulated by the AMP-activated protein kinase (AMPK) [58] and mechanistic target of rapamycin complex 1 (mTORC1) [59], respectively (Fig. 2). mTORC1 is a master regulator of cell growth, which, in response to nutrient availability and growth factor stimuli, promotes cellular anabolism, but inhibits catabolic pathways, including autophagy. Under nutrient-rich conditions, mTORC1 blocks autophagy induction by suppressing the ULK1 complex via the phosphorylation of ULK1/2 or ATG13 [60]. When nutrients become limiting and cellular ATP levels decrease, AMPK is activated, while mTORC1 is inhibited, leading to the activation of ULK1 complex and subsequent induction of autophagy [58]. Thus, the ULK1 complex integrates signals from the two major sensors of energy stress and nutrient status, playing a central role in autophagy induction.

**Fig. 3:**
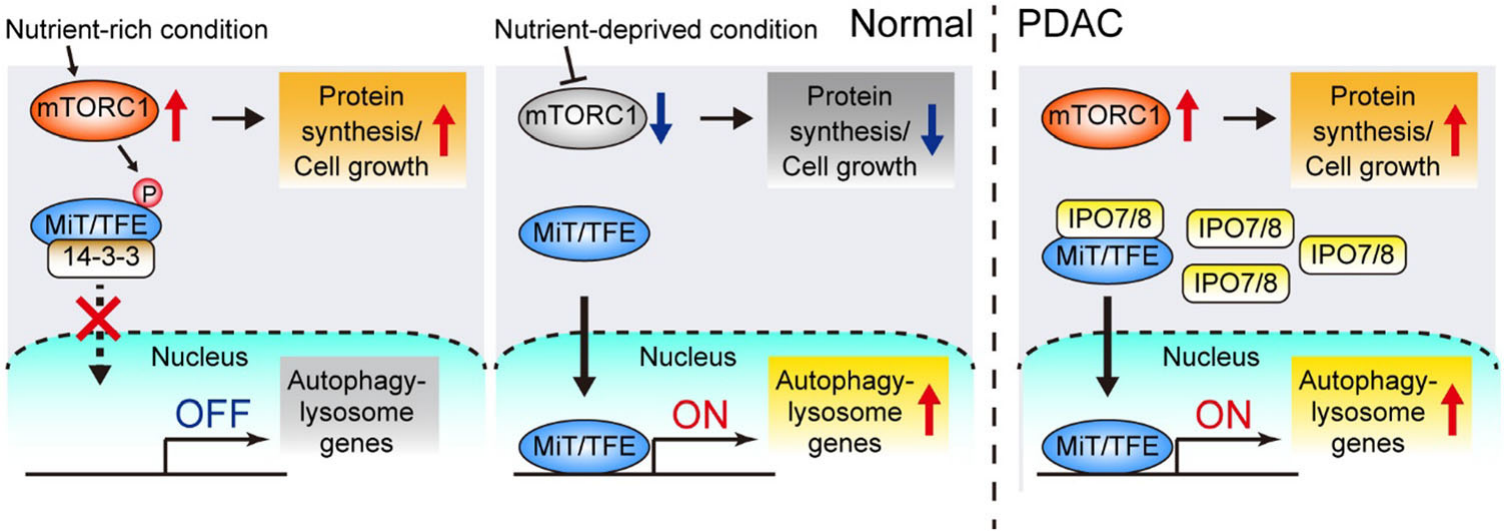
Mechanisms of constitutively high autophagy-lysosome activity in PDAC. The MiT/TFE family transcription factors are critical regulators of autophagy-lysosome genes. (*Left*) Under nutrient-rich conditions, MiT/TFE proteins are repressed by mTORC1 via phosphorylation and remain in the cytosol. (*Middle*) Upon starvation, mTORC1 is inactivated and MiT/TFE proteins enter the nucleus, where they activate autophagy-lysosome gene transcription. (*Right*) PDAC cells overexpress the nucleocytoplasmic transporter, importin (IPO)-7/8, to facilitate the nuclear translocation of MiT/TFE, thereby decoupling MiT/TFE from mTORC1-mediated suppression and upregulating autophagy-lysosome gene expression. Consequently, PDAC maintains constitutively high basal autophagy/lysosome activity under sustained mTORC1 activity, thus simultaneously employing the catabolic process mediated by the autophagy/lysosomal pathway and the anabolic process driven by mTOR signaling to maximize their proliferation [64]

In addition to this kinase-dependent regulation of acute autophagy induction, transcriptional programs are also utilized to maintain the increased activity of the autophagy-lysosome pathway. The microphthalmia/transcription factor E (MiT/TFE) family of transcription factors (MITF, TFE3, and TFEB) controls transcriptional programs for autophagy and lysosome biogenesis via the coordinated upregulation of autophagy and lysosome genes [61–63]. Under nutrient-rich conditions, MiT/TFE proteins are phosphorylated by mTORC1, leading to interactions with 14–3-3 and cytoplasmic retention. Under starved conditions, mTORC1 is inactivated and MiT/TFE factors undergo nuclear translocation, where they activate their target genes via direct binding to a consensus sequence, termed as the coordinated lysosomal expression and regulation (CLEAR) sequence [62] (Fig. 3).

Importantly, some cancers, including PDAC, exhibit elevated autophagy and lysosome biogenesis to achieve efficient nutrient recycling through degradation of lysosomal cargo, while simultaneously maintaining high mTORC1 activity to drive cellular anabolism and proliferation. Perera et al. found that PDAC cells show constitutive nuclear localization of MiT/TFE proteins, regardless of the nutrient status [64]. They showed that PDAC cells overexpress both MiT/TFE proteins and the nuclear import proteins, importin (IPO)-7 and IPO8, which facilitates the nuclear translocation of MiT/TFE factors, though the mechanism by which increased IPO7/8 levels can decouple MiT/TFE factors from mTORC1-mediated regulation in PDAC cells remains unknown. Consequently, PDAC cells exhibit constitutive activation of autophagy-lysosome transcriptional programs regardless of nutrient availability and mTORC1 activity. Importantly, depletion of MiT/TFE proteins in PDAC cells abrogates the autophagy-lysosomal function and impairs tumor growth, highlighting the critical role of this transcriptional program in the survival and growth of PDAC cells [64](Fig. 3).

Wong et al. discovered active dephosphorylation of ULK1 by protein phosphatase 2A (PP2A) as another mechanism for elevated autophagy flux in PDAC [70]. mTORC1 inhibits autophagy via the phosphorylation of ULK1 at multiple sites, including S637 and S757 [71, 72](Fig. 2). The authors found that the PP2A–B55α complex dephosphorylated S637 on ULK1 and counteracted mTORC1-mediated autophagy suppression in PDAC. They also showed that increased PP2A phosphatase activity in PDAC cells drives increased basal autophagy. Importantly, this process did not impair mTORC1 activity. Together, these studies highlight the mechanisms by which PDAC cells maintain high basal autophagy and increased lysosomal biogenesis to meet the metabolic demands, while simultaneously exploiting mTORC1 activity to maximize protein synthesis and cell proliferation.

How do PDAC cells maintain the lysosomal membrane integrity to cope with the huge influx of cargo into lysosomes? Gupta et al. used a proteomics-based approach and discovered that Myoferlin, a plasma membrane repair factor, is enriched in the lysosomal membranes of PDAC cells, maintaining their integrity and function [65]. Importantly, Myoferlin is upregulated in human PDAC, which is associated with poor patient survival, and Myoferlin ablation abrogates tumor growth in murine PDAC models, highlighting the crucial role of the autophagy-lysosomal pathway in PDAC progression [65].

### Host autophagy supports tumor metabolism

Although initial studies mainly focused on the role of autophagy in cancer cells, recent studies have demonstrated that host autophagy (autophagy in normal host cells) also contributes to tumor progression. Karsli–Uzunbas et al. used Kras^G12D^- and Trp53^−/−^-driven murine lung cancer models and showed that the conditional knockout of Atg7 in the whole body (in host and cancer cells) leads to the substantial regression of established tumors, which, surprisingly, was even greater than Atg7-knockout in cancer cells alone, suggesting that both host autophagy and tumor-specific autophagy support tumor growth [26]. Similar results were obtained in different models, including GEMM of non-small lung cancer, melanoma, PDAC [38, 55, 56], and RasG12V-driven tumors in *Drosophila melanogaster* [66], where autophagy-proficient cancer cells were transplanted into autophagy-proficient or autophagy-deficient animals. In these studies, tumor growth was impaired when cells were transplanted into autophagy-deficient hosts, confirming the tumor-promoting role of host autophagy.

**Fig. 4:**
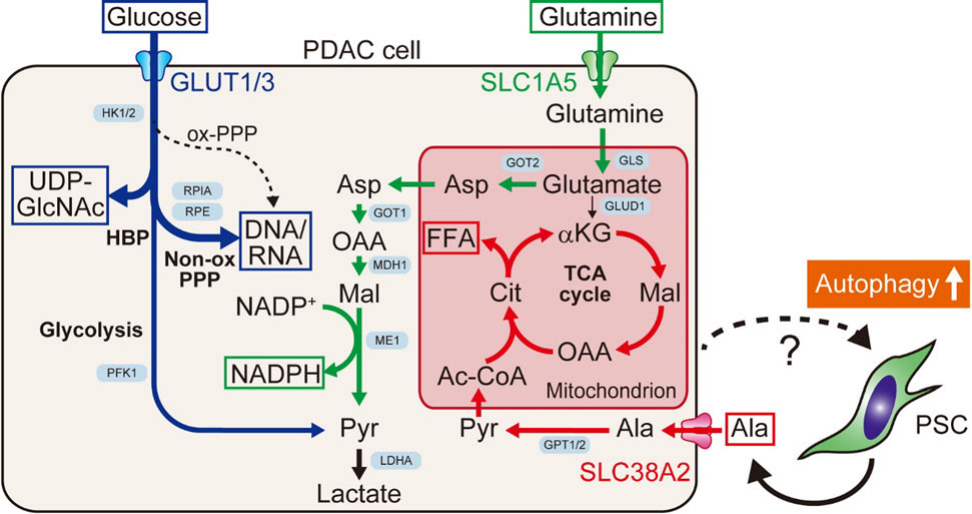
Autophagy in non-cancer cells supports unique metabolic properties in PDAC. In response to stimuli from PDAC cells, PSCs become activated and secrete alanine in an autophagy-dependent manner. This PSC-derived alanine is taken up by PDAC cells and used to fuel the TCA cycle and biosynthesis of free fatty acids (FFAs)(red), allowing PDAC cells to use glucose and glutamine primarily to generate nucleic acids [69](blue) and NADPH for redox control [70](green). *Ac-CoA* acetyl-CoA, *Ala* alanine, *aKG* α-ketoglutarate, *Asp* aspartate, *Cit* citrate, *GLUD1* glutamate dehydrogenase 1, *GLS* glutaminase, *GLUT1/3* glucose transporter 1/3, *GOT1/2* aspartate aminotransferase ½, *GPT1/2* alanine aminotransferase ½, *HBP* hexosamine biosynthesis pathway, *HK1/2* hexokinase ½, *LDHA* lactate dehydrogenase A, *MDH1* malate dehydrogenase 1, *ME1* malic enzyme 1, *Mal* malate, *Non-ox PPP* oxidative branch of pentose phosphate pathway, *OAA* oxaloacetic acid, *ox-PPP* oxidative branch of pentose phosphate pathway, *PFK1* phosphofructokinase 1, *Pyr* pyruvate, *RPE* ribulose-phosphate 3-epimerase, *RPIA* ribose-5-phosphate isomerase, *SLC1A5* solute carrier family 1 member 5, *TCA* tricarboxylic acid, *UDP-GlcNAc* uridine diphosphate *N*-acetylglucosamine

How does host autophagy support tumor growth? Sousa et al. showed that pancreatic stellate cells (PSCs), a major source of cancer-associated fibroblasts (CAFs) in PDAC tumor stroma [67], secrete alanine in an autophagy-dependent manner [68]. This PSC-derived alanine is taken up by PDAC cells to fuel the tricarboxylic acid cycle and for the de novo synthesis of non-essential amino acids and free fatty acids, allowing PDAC cells to use glucose and glutamine, two key nutrients that are scarce in the PDAC TME [11, 12], for other biosynthesis pathways, such as nucleic acid biogenesis [69] and production of NADPH to maintain redox balance [70]. Thus, autophagy-dependent alanine secretion from PSCs plays a pivotal role in supporting the unique metabolism of PDAC cells (Fig. 4). A follow-up study revealed the detailed mechanism of this alanine crosstalk, where PSCs use SLC1A4 to secrete alanine, and PDAC cells upregulate SLC38A2 to take up alanine [71]. Notably, SLC38A2 ablation causes a metabolic crisis in PDAC cells, but not in normal cells, leading to substantial tumor regression, highlighting the crucial role of PSC-derived alanine in supporting the unique metabolic rewiring in PDAC cells [71]. Similarly, host autophagy supports tumor growth by sustaining circulating arginine levels, especially in cancer types that lack arginine synthase expression and require exogenous arginine for survival [55]. Depletion of *Atg5* or *Atg7* in host cells leads to arginase 1 secretion, an arginine-degrading enzyme, from the liver into the circulation, which degrades and lowers serum arginine levels, eventually impairing the growth of arginine auxotrophic tumors [55].

How do cancer cells induce autophagy in healthy host cells? Katheder et al. used an RAS-driven tumor model in *D. melanogaster* and discovered that tumor cells trigger autophagy in neighboring cells and distant organs, which fuels tumor growth and metastasis by providing amino acids [66]. The authors further identified tumor-cell-derived ROS as key mediators regulating host autophagy, although factors other than ROS may also be involved in autophagy induction in host cells, particularly in distant organs.

In addition to alanine, CAFs also secrete other metabolites, such as lipids, which support biomass production in PDAC cells [72], and pyrimidine, a deoxycytidine that blunts the efficacy of the nucleotide analog, gemcitabine [73, 74]. CAFs also secrete various cytokines and chemokines, including C-X-C motif chemokine ligand (CXCL)-12, interleukin (IL)-6, leukemia inhibitory factor (LIF), and Netrin-G1, to promote PDAC progression [75–77]. Notably, the activation of PSCs into tumor-promoting CAFs requires the upregulation of autophagy [68, 78] as well as other stimuli, such as vitamin D receptor (VDR) signaling [79]. Taken together, these data suggest that targeting autophagy in host cells, including PSCs/CAFs, represents an attractive therapeutic strategy for PDAC and other cancers.

### Host autophagy promotes tumor immune tolerance

To date, many studies have demonstrated that host autophagy supports tumor metabolism by providing essential nutrients. Recent studies have also shed light on the role of host autophagy as a negative regulator of anti-tumor immunity, where the inhibition of autophagy in host cells enhances anti-tumor immune responses and suppresses tumor growth.

#### Autophagy in the liver

Poillet-Perez et al. used murine melanomas that carry a high tumor mutational burden and demonstrated that the loss of autophagy in normal host cells, especially in the liver, promotes T cell infiltration into tumors, limiting tumor growth in a T cell-dependent manner [56]. Mechanistically, the authors showed that host autophagy suppresses anti-tumor T cell responses by enhancing the regulatory T cell (Treg) functions and suppressing interferon-g signaling, although the exact mediators remain to be elucidated.

#### Autophagy in T cells

DeVorkin et al. reported that loss of autophagy in CD8^+^ T cells enhances their tumor-cell-killing effect and impairs tumor growth [80]. Mechanistically, autophagy inhibition shifts T cells to a more glycolytic state and reduces the production of S-adenosylmethionine (SAM), an important methyl-donor, leading to global changes in epigenetic modifications, including the loss of H3K27me3 and gain of H3K4me3 marks. These epigenetic alterations transcriptionally reprogram CD8^+^ T cells to an effector memory state, enabling the efficient killing of cancer cells.

#### Autophagy-related pathways in myeloid cells

Other autophagy-related pathways in host cells have also been shown to regulate the anti-tumor immune responses. LC3-associated phagocytosis (LAP) is a form of phagocytosis used by macrophages to clear pathogens or dead cells and regulate their immune responses [81]. LAP utilizes many core components of the autophagy pathway, including VPS34, BECN1, ATG3, ATG5, ATG7, and ATG12. Cunha et al. showed that the impairment of LAP, but not autophagy, in tumor-associated macrophages transforms them into a tumor suppressive M1 subtype, which promotes anti-tumor T cell responses and impairs tumor growth [82]. Similarly, Chen et al. showed that the lysosomal inhibitor chloroquine (CQ) impairs tumor growth by reprograming macrophages from the tumor-promoting M2 subtype to the tumor-suppressive M1 subtype and enhancing the anti-tumor T cell response. Together, these studies revealed that autophagy and lysosome-related activity in host cells promote tumor immune tolerance, representing attractive therapeutic targets to enhance the anti-tumor immunity.

### Autophagy facilitates immune evasion across cancer types

PDAC is one of the tumor types most resistant to ICB. Apart from rare cases with microsatellite instability-high (MSI-High) or mismatch repair deficiency (dMMR) [2–4], the vast majority of patients with PDAC have failed to benefit from ICB [83–86]. This resistance of PDAC to ICB has been ascribed to multiple factors, including immune suppressive TME, limited number of CD8^+^ T cells infiltrating the tumor, or low tumor mutational burden [87], features that have been verified in GEMMs of PDAC [88]. However, more recent evidence has revealed that human PDAC tumors have a varying number of CD8^+^ T cells with considerable intratumoral heterogeneity and sometimes harbor antigens that can stimulate potent T cell immunity [89–92], raising the possibility that there may be other undiscovered factors mediating immunotherapy resistance.

**Fig. 5:**
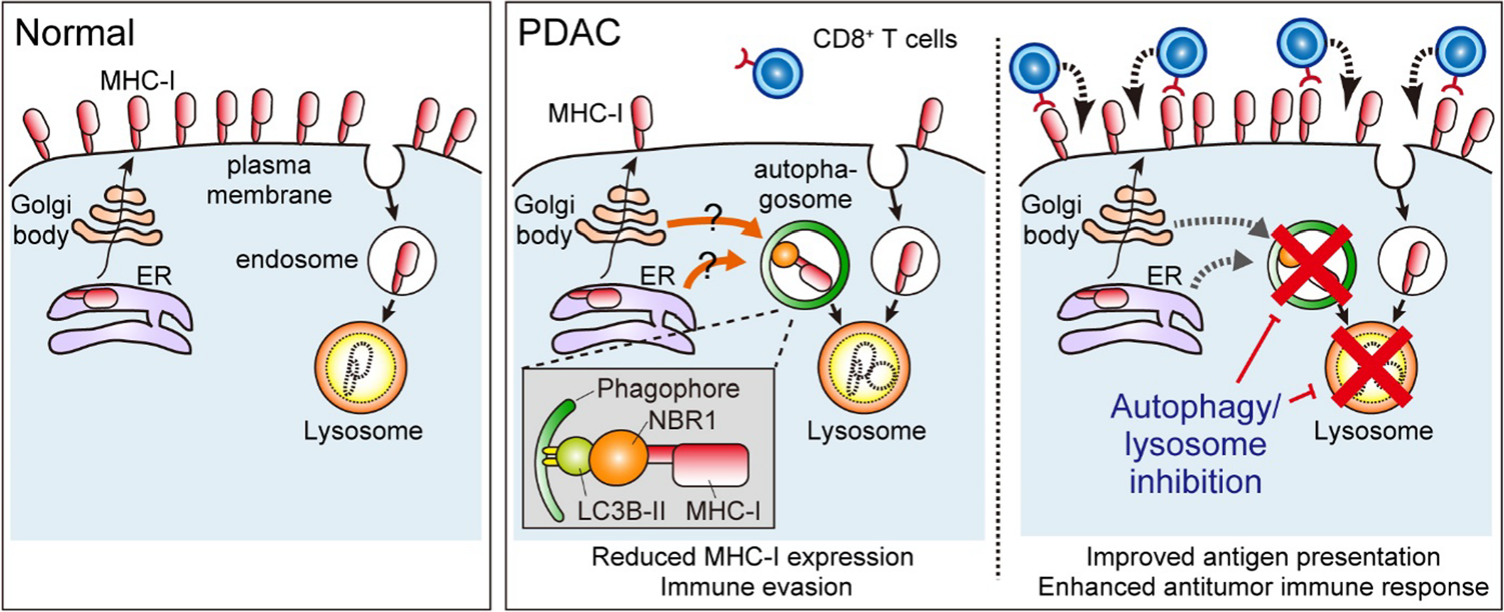
Selective autophagy of MHC-I promotes immune evasion of PDAC. (*Left*) In normal cells, major histocompatibility complex class I (MHC-I) is localized on the plasma membrane, where it presents endogenous antigens to CD8.^+^ T cells. (*Middle*) In PDAC cells, MHC-I is actively targeted for lysosomal degradation through NBR1-mediated selective autophagy, leading to reduced MHC-I levels on the plasma membrane, thereby facilitating immune evasion. (*Right*) Importantly, autophagy or lysosome inhibition restores MHC-I expression, leading to enhanced anti-tumor T cell immunity and improved response to ICB [101]. *ER* endoplasmic reticulum

Major histocompatibility complex class I (MHC-I) is a key component of the acquired immune system, in which cells present endogenous peptides to CD8^+^ T cells. If virus-infected cells or cancer cells present peptides derived from non-self proteins, such as viral components or mutated proteins, CD8^+^ T cells recognize these non-self peptides with their T-cell receptors (TCRs) and eliminate these cells. Unsurprisingly, MHC-I expression or other antigen presentation machinery, which is indispensable for CD8^+^ T cell-mediated tumor-cell killing, is frequently dysregulated or lost in various cancer types, resulting in resistance to ICB [93–96]. In both human and mouse PDAC tumors, MHC-I expression is downregulated, which is particularly evident in liver metastasis [97, 98]. However, unlike other types of cancer, mutations in MHC-I are rare in PDAC [99]. A recent study led by a joint team of the Kimmelman and Perera labs demonstrated that MHC-I is actively degraded via autophagy in PDAC, thereby reducing cell surface MHC-I levels and facilitating immune evasion [100, 101]. The team also identified NBR1 as an autophagy cargo receptor that enables the selective targeting and degradation of MHC-I molecules in autophagosomes and lysosomes. Importantly, genetic and pharmacological inhibition of autophagy/lysosome activity restores cell surface MHC-I expression, increases T-cell infiltration into tumor, and sensitizes PDAC to ICB in murine models [100, 101] (Fig. 5). In line with this, a recent clinical trial using HCQ and gemcitabine plus nabpaclitaxel (GnP), a standard-of-care chemotherapy for PDAC [102], as neoadjuvant chemotherapy (NCT01978184), showed that the addition of HCQ increased the number of tumor-infiltrating CD8^+^ T cells [103, 104]. Additionally, a recent study identified tumor-cell-derived progranulin (PGRN) as a driver of autophagy-dependent MHC-I degradation and subsequent immune evasion [105]. The authors used surgically resected human PDAC tumors and showed that increased PGRN expression in PDAC cells is associated with lower MHC-I expression and fewer CD8^+^ T cell infiltration as well as shorter patient survival. Importantly, inhibition of PGRN by a neutralizing antibody blocked this autophagy-mediated MHC-I degradation, restoring MHC-I expression in PDAC cells, and enhancing anti-tumor T cell responses in the murine PDAC model [105]. Together, these studies support the role of autophagy as a negative regulator of MHC-I-mediated antigen presentation in PDAC, highlighting that autophagy inhibition is a promising strategy for sensitizing PDAC to ICB.

Accumulating evidence supports the role of autophagy as a negative regulator of anti-tumor immunity in various types of cancer. Notably, multiple studies using unbiased genome-wide CRISPR knockout screens have identified autophagy as a key cancer-cell intrinsic mechanism that drives immune evasion across cancer types [106–108] (Table 1). The major mechanisms of autophagy-mediated immune evasion include impaired antigen presentation [100, 101, 105, 109], downregulation of T cell-attracting chemokines, such as CXCL9 and CXCL10 [110–113], and resistance to interferon-γ and TNF-α, key inflammatory mediators that are released from cytotoxic CD8^+^ T cells [106–108]. In addition to autophagy in cancer cells, host autophagy and other autophagy-related pathways in host cells contribute to tumor immune evasion [56, 80, 82, 114]. Based on these compelling preclinical results, several ongoing clinical trials are combining autophagy inhibition and ICB to treat PDAC (Table 2).

**Table 1:** Preclinical evidence showing enhanced anti-tumor immune response upon autophagy inhibition in cancer cells

Cancer types	Study methods	Comments	Refs
Pancreatic cancer	Short hairpin RNA (shRNA)-mediated knock down of autophagy-related (ATG) genes; Overexpression of Atg4B^C74A^; Murine syngeneic tumor transplantation models	MHC-I is degraded via NBR-I-mediated selective autophagy in PDAC cells, promoting their immune evasion. Autophagy-inhibition restores cell surface MHC-I expression and sensitizes PDAC to immune checkpoint blockade (ICB)	[100, 101]
Pancreatic cancer	Blocking antibody against progranulin (PGRN); Genetically engineered mouse model (GEMM) of PDAC; Murine syngeneic tumor transplantation models	Tumor-derived PGRN drives autophagy-dependent MHC-I degradation in PDAC. Increased PGRN expression in PDAC cells is associated with low MHC-I expression, decreased CD8^+^ T cell infiltration, and decreased patient survival. PGRN blockade restores MHC-I expression in PDAC cells, improving anti-tumor T cell responses	[105]
Pancreatic cancer	Murine syngeneic tumor transplantation model; Cobimetinib (MEK inhibitor), mefloquine (MFQ, lysosome inhibitor)	Dual inhibition of MEK and autophagy in PDAC cells activates the STING/type I interferon (IFN) pathway, which polarizes tumor-associated macrophages into tumor-suppressive M1-like subtype. Addition of agonistic antibody to CD40 further augments this autophagy/MEK inhibition-mediated immune response, eliciting robust T cell responses	[124]
Melanoma; Breast cancer; Renal cell carcinoma; Colorectal cancer	Genome-wide CRISPR knockout screen; Murine syngeneic tumor transplantation models	Autophagy pathway is identified as a top-ranked mediator of immune evasion by cancer cells. Mechanistically, autophagy in cancer cells confers resistance to IFN-g and TNF-a that are released from cytotoxic T lymphocytes	[106–108]
Melanoma; Colorectal cancer	Vps34 inhibitor, shRNA to Vps34; Murine syngeneic tumor transplantation models	Vps34 inhibition/knockdown increases cancer cell secretion of CXCL5 and CXCL10, which recruit natural killer, CD4^+^ T, and CD8^+^ T cells into tumors, leading to tumor regression and improved response to ICB	[111]
Melanoma; Colorectal cancer	CRISPR-mediated knockout of Atg7; Murine syngeneic tumor transplantation models	Upon syngeneic transplantation, Atg7-knockout mouse cancer cells stimulated enhanced anti-tumor immune response, which was mediated by CD8^+^ T cells. This enhanced immune response likely depends on the immunogenicity of the cancer cells	[136]
Breast cancer	CRISPR-mediated knockout of Atg5 and Atg7; Murine syngeneic tumor transplantation models	Upon radiation, autophagy-deficient cancer cells accumulate mitochondrial DNA in the cytosol, which stimulates the cGAS-STING pathway and induces type I IFN secretion, resulting in improved response to ICB and tumor regression in non-irradiated and irradiated lesions (Abscopal effect)	[112]
Non small cell lung cancer (NSCLC)	MRT68921 (ULK1 inhibitor) or CQ; overexpression of Atg4B^C74A^; Murine syngeneic tumor transplantation models	Inactivating mutations in serine/threonine kinase 11 (STK11/LKB1) are found in 20% of NSCLC cases and associated with poor response to ICB. LKB1 deficiency increases mutational burden and suppresses antigen processing and presentation, which are associated with compromised immunoproteasome activity and elevated autophagy flux. Inhibition of autophagy by an ULK1 inhibitor restores antigen presentation and sensitizes LKB1-mutant NSCLC to programmed death 1 (PD1) blockade therapy	[109]
Prostate cancer	PIKfyve inhibitor ESK981; Murine syngeneic tumor transplantation models	A multi kinase inhibitor, ESK981, showed potent anti-tumor effects on castration-resistant prostate cancer (CRPC) tumor in a CD8^+^ T cell-dependent manner. Mechanistically, ESK981 targets the lipid kinase, PIKfyve, and blocks autophagy flux in CRPC cells, which results in increased release of T-cell chemoattractants, CXCL10 and CCL2, thereby enhancing T cell infiltration into tumors and potentiating ICB response	[113]

**Table 2:** Clinical trials targeting autophagy/lysosome in pancreatic cancer

Disease	Interventions	Results	Study phase	Refs
Previously treated, Meatastatic	HCQ	0% response	II	NCT01273805 [126]
Borderline resectable	Gem + HCQ (neoadjuvant)	CA19-9 decrease in 61% cases; R0 resection in 77% cases; Prolonged OS in patients with significant autophagy inhibition in peripheral blood cells (34.83 vs 10.83 months, p < 0.05)	I/II	NCT01128296 [137]
Metastatic or unresectable	Gem + CQ	Median PFS, 4 months; Median OS, 7.6 months	I	NCT01777477 [138]
Previously untreated, metastatic or Advanced	GnP ± HCQ	Improved RR in GnP with HCQ than in GnP without HCQ (38.2 vs 21.1%); no difference in OS and PFS	I/II	NCT01506973 [127]
Resectable or borderline resectable	GnP ± HCQ (neoadjuvant)	Addition of HCQ to GnP improved histopathologic RR (p = 0.00016) and tumor infiltrating CD8 + T cells. Intratumoral αβ T cell receptor clonality was associated with CA 19–9 response and prolonged OS	II	NCT01978184 [103] [104]
Previously untreated, Metastatic	GnP + HCQ + Ipilimumab (anti-CTLA4 antibody)	Ongoing	I	NCT04787991
Gastrointestinal cancer (pancreatic and colorectal cancer)	Cobimetinib (MEK inhibitor) + Atezolizumab (anti-PDL1 antibody) + HCQ	Ongoing	I/II	NCT04214418
Advanced	Trametinib (MEK inhibitor) + HCQ	Ongoing	I	NCT03825289
Metastatic	Binimetinib (MEK inhibitor) + HCQ	Ongoing	I	NCT04132505
Advanced	Ulixertinib (ERK inhibitor) + HCQ	Ongoing	I	NCT04145297
Metastatic	LY3214996 ± HCQ (ERK inhibitor)	Ongoing	II	NCT04386057
Advanced pancreatic cancer	mFOLFIRINOX + HCQ + Chlorphenesin Carbamate	Ongoing	I	NCT05083780
Advanced or metastatic	Gem + nabpaclitaxel ± Paricalcitol (Vitamin D receptor agonist) plus HCQ (GnP ± PH)	Ongoing	II	NCT04524702

*Gem* gemcitabine, *GnP* gemcitabine + nabpaclitaxel, *HCQ* hydroxychloroquine, *OS* overall survival, *PFS* progression-free survival, *RR* response rate, *R0* resection, complete tumor removal with negative resection margins

### Autophagy as a driver of therapeutic resistance

Autophagy has been implicated in resistance to treatment [115–117]. For example, the mitogen-activated protein kinase (MAPK) pathway, a downstream pathway of KRAS, plays a central role in the initiation and progression of cancers driven by RAS or RAF mutations, including PDAC, colorectal cancer, lung cancer, and melanoma [118]. Therapeutic approaches targeting major components of the RAS–MAPK pathway, such as RAF, MEK, and ERK, have been hampered by the inevitable emergence of acquired resistance [119]. One such mechanism is increased autophagy flux, which was initially shown in a study where PDAC cells that survived oncogenic KRAS ablation exhibited elevated autophagy [120]. Consistently, several groups have found that the RAS–MAPK pathway inhibition increases autophagy flux in multiple cancer types, including PDAC, and that combined inhibition of the RAS–MAPK pathway and autophagy exerts synergistic anti-tumor effects [121–123]. Notably, the combination of the MEK inhibitor, trametinib, and HCQ resulted in drastic tumor shrinkage in a patient with metastatic PDAC [122]. More recently, Jiang et al. reported that dual inhibition of MEK and autophagy, but not either treatment alone, activates the STING/type I interferon pathway in PDAC cells, which reprograms tumor-associated macrophages into a tumor-suppressive M1-like subtype [124]. Importantly, the addition of an agonistic antibody to CD40, a costimulatory molecule that licenses and activates antigen-presenting cells upon ligand engagement [125], further augments this autophagy/MEK inhibition-mediated immune response, eliciting robust T-cell responses in triple therapy [124]. Based on these promising preclinical data, multiple clinical trials are underway to test the efficacy of the combined treatment with HCQ and MEK/ERK inhibitors (Table 2).

### Clinical trials targeting autophagy in PDAC

Currently, lysosomal inhibitors, CQ and HCQ, which have long been used as antimalarial drugs, are the only clinically available agents to block autophagy. Both compounds have been used in several clinical trials to target various cancers, including PDAC [116]. Although HCQ showed only minimal activity when used as monotherapy [126], HCQ plus GnP significantly improved the response rates compared with GnP alone (21% vs. 38%, *p* = 0.047) [127], showed an improved pathological response, and increased immune cell infiltration [103, 104](NCT01978184), further confirming the role of autophagy as a key resistance mechanism against therapeutic treatment. Based on these findings, multiple clinical trials are ongoing to test the efficacy of combining autophagy/lysosome inhibitors (mainly HCQ) with conventional chemotherapy, targeted therapy, and ICB (Table 2).

### Acquired resistance to autophagy inhibition

Autophagy inhibition is a promissing therapeutic option; hence, the emergence of acquired resistance to autophagy inhibition is an obstacle that must be overcome for effective treatment. Recent studies have identified NRF2 upregulation as a major mechanism by which cancer cells circumvent autophagy inhibition [128, 129]. Towers et al. found that cancer cells that have adapted to autophagy deficiency upregulate NRF2 signaling, a master regulator of cellular stress responses [44], thereby compensating for the impaired ubiquitin proteasome system function and ER stress caused by autophagy deficiency [128]. Su et al. showed that the inhibition of autophagy in PDAC cells upregulates macropinocytosis to enhance extracellular nutrient scavenging, which is mediated by NRF2-driven transcriptional upregulation of macropinocytosis-related genes [129]. In both studies, compensatory NRF2 signaling following autophagy inhibition was induced by the accumulation of the autophagy cargo receptor protein, p62, which blocks Keap1-mediated degradation of NRF2 [45, 46, 130], thereby derepressing NRF2 activity [128, 129]. Importantly, these studies demonstrated that combining proteasome inhibition [128] or macropinocytosis inhibition [129] is effective in overcoming resistance to autophagy inhibition in PDAC.

## Future perspectives

Despite compelling preclinical evidence showing the efficacy of autophagy inhibition as a therapeutic strategy against PDAC, clinical trials using the lysosomal inhibitor, CQ/HCQ, have shown only minimal to modest impact on patient prognosis (Table 2). Given the unfavorable pharmacokinetics of these drugs in vivo [131], more potent inhibitors are required to improve the patient outcome. Additionally, to accelerate the development of novel inhibitors and identify patients likely to benefit from autophagy inhibitors, it is crucial to develop reliable biomarkers, preferably less invasive ones that enable repeated measurements, to facilitate the monitoring of autophagy activity in human patients [132]. Another concern in the use of CQ/HCQ to block autophagy is the fact that CQ/HCQ is not specific to autophagy: CQ/HCQ inhibits lysosomal acidification and impairs all lysosome-related pathways, including endocytosis, macropinocytosis, LAP [81], and LC3-associated endocytosis (LANDO), a process recently shown to be involved in b-amyloid clearance and neurodegeneration prevention [133]. However, given the tumor-promoting roles of macropinocytosis in PDAC cells [129] and LAP in host cells [82], inhibiting all lysosome-related pathways using CQ/HCQ may be more efficacious than autophagy-specific inhibition in PDAC treatment. More potent and specific autophagy inhibitors are required, both as clinical drug candidates and research tools.

Another unanswered question regarding the use of autophagy inhibition as a therapeutic strategy is whether autophagy should be inhibited only in cancer cells or systemically in the whole body, although there is no autophagy-lysosome inhibitor that can specifically target only cancer cells. Given the tumor-promoting roles of host autophagy and tumor-cell intrinsic autophagy, blocking autophagy in both cancer and host cells may be more efficacious. However, considering the multifaceted functions of autophagy in normal physiology, it is important to determine the effect of autophagy inhibition on normal cells in the body. In particular, when inhibiting autophagy to enhance the anti-tumor immune response, the effects of autophagy inhibition on respective immune cells and anti-tumor immune response have to be carefully determined, as previous studies have shown both favorable [134] and unfavorable [135] effects of autophagy inhibition on different types of immune cells. To this end, a detailed analysis of the surgically resected tumors that received preoperative treatment regimens involving autophagy inhibition may be of great help to expand our knowledge [131].

## Abbreviations

TME: Tumor microenvironment
PDAC: Pancreatic ductal adenocarcinoma
GEMM: Genetically engineered mouse model
ICB: Immune checkpoint blockade
CQ: Chloroquine
HCQ: Hydroxychloroquine
